# A GDF11/myostatin inhibitor, GDF11 propeptide-Fc, increases skeletal muscle mass and improves muscle strength in dystrophic mdx mice

**DOI:** 10.1186/s13395-019-0197-y

**Published:** 2019-05-27

**Authors:** Quan Jin, Chunping Qiao, Jianbin Li, Bin Xiao, Juan Li, Xiao Xiao

**Affiliations:** Division of Pharmacoengineering and Molecular Pharmaceutics, Eshelman School of Pharmacy, University of North Carolina, Chapel Hill, NC USA

**Keywords:** GDF11, Myostatin, Hypertrophy, mdx, Duchenne muscular dystrophy, AAV, Gene delivery, Gene therapy

## Abstract

**Background:**

Growth differentiation factor 11 (GDF11) is a member of the transforming growth factor β superfamily. The GDF11 propeptide, which is derived from the GDF11 precursor protein, blocks the activity of GDF11 and its homolog, myostatin, which are both potent inhibitors of muscle growth. Thus, treatment with GDF11 propeptide may be a potential therapeutic strategy for diseases associated with muscle atrophy like sarcopenia and the muscular dystrophies. Here, we evaluate the impact of GDF11 propeptide-Fc (GDF11PRO-Fc) gene delivery on skeletal muscle in normal and dystrophic adult mice.

**Methods:**

A pull-down assay was used to obtain physical confirmation of a protein-protein interaction between GDF11PRO-Fc and GDF11 or myostatin. Next, differentiated C2C12 myotubes were treated with AAV6-GDF11PRO-Fc and challenged with GDF11 or myostatin to determine if GDF11PRO-Fc could block GDF11/myostatin-induced myotube atrophy. Localized expression of GDF11PRO-Fc was evaluated via a unilateral intramuscular injection of AAV9-GDF11PRO-Fc into the hindlimb of C57BL/6J mice. In mdx mice, intravenous injection of AAV9-GDF11PRO-Fc was used to achieve systemic expression. The impact of GDF11PRO-Fc on muscle mass, function, and pathological features were assessed.

**Results:**

GDF11PRO-Fc was observed to bind both GDF11 and myostatin. In C2C12 myotubes, expression of GDF11PRO-Fc was able to mitigate GDF11/myostatin-induced atrophy. Following intramuscular injection in C57BL/6J mice, increased grip strength and localized muscle hypertrophy were observed in the injected hindlimb after 10 weeks. In mdx mice, systemic expression of GDF11PRO-Fc resulted in skeletal muscle hypertrophy without a significant change in cardiac mass after 12 weeks. In addition, grip strength and rotarod latency time were improved. Intramuscular fibrosis was also reduced in treated mdx mice; however, there was no change seen in central nucleation, membrane permeability to serum IgG or serum creatine kinase levels.

**Conclusions:**

GDF11PRO-Fc induces skeletal muscle hypertrophy and improvements in muscle strength via inhibition of GDF11/myostatin signaling. However, GDF11PRO-Fc does not significantly improve the dystrophic pathology in mdx mice.

**Electronic supplementary material:**

The online version of this article (10.1186/s13395-019-0197-y) contains supplementary material, which is available to authorized users.

## Background

Growth differentiation factor 11 (GDF11) is a member of the transforming growth factor beta (TGF-β) superfamily. It was first identified as a critical factor in embryogenesis, with important roles in axial patterning, neurogenesis, and organogenesis [1–4]. GDF11 belongs to the same subgroup in the TGF-β superfamily as myostatin (MSTN; GDF8), and the two factors share 90% homology in their mature domain protein sequences. Both GDF11 and MSTN signal through activin type II receptors (ActRII). In the canonical pathway, GDF11 binding at ActRII initiates heterodimerization of the ActRII with a type I activin receptor (ALK4/5) and phosphorylation of the SMAD2/3 transcription factor. Phosphorylated SMAD2/3 then recruits SMAD4 and the SMAD2/3/4 complex translocates to the nucleus where it mediates changes in gene expression [5–8]. In skeletal muscle, activation of the TGF-β-ActRII-SMAD2/3 signaling axis is strongly associated with inhibition of muscle growth [9].

GDF11 has been reported to have anti-aging benefits in the heart, brain, and skeletal muscle [10–12]. However, these findings remain controversial, especially those regarding the capacity of GDF11 treatment to rejuvenate aged skeletal muscle [13]. Several independent groups, including ours, have shown that GDF11 induces substantial skeletal muscle atrophy and inhibition of skeletal muscle growth using a variety of approaches in vivo, including direct administration of recombinant GDF11 (rGDF11) protein, GDF11 gene delivery and injection of GDF11-secreting Chinese hamster ovary (CHO) cells [14–17]. In addition, increased intramuscular fibrosis was observed in dystrophic mdx mice treated with daily intraperitoneal injections of rGDF11 [18].

The GDF11 propeptide is a natural inhibitor of GDF11 derived from the GDF11 precursor protein. Like other TGF-β ligands, GDF11 is initially translated as an inactive precursor protein homodimer consisting of a signal peptide, N-terminal propeptide, and C-terminal mature domain. An initial proteolytic step carried out by a furin-like protease cleaves the N-terminal propeptide from the C-terminal mature domain. After this cleavage step, the propeptide remains non-covalently associated with the mature domain in an inactive latent complex. A subsequent proteolytic step mediated by a bone morphogenetic protein 1 (BMP1)/Tolloid (TLD)-like metalloproteinase cleaves the N-terminal propeptide and releases the mature GDF11 dimer [19, 20]. Although the mature domains of GDF11 and MSTN are highly homologous, their propeptides share only 49% homology in their protein sequences. It has been previously demonstrated that the GDF11 propeptide inhibits both GDF11 and MSTN activity in vitro [21]. However, the effects of exogenous GDF11 propeptide delivery in vivo have not been well-characterized.

Recombinant adeno-associated virus (AAV) vectors, owing to their exceptional safety and efficacy profile, are frequently used for in vitro and in vivo gene delivery applications [22]. AAV vectors transduce both dividing and non-dividing cells, and vector genomes are predominantly converted into extrachromosomal episomal DNA in the nucleus [23]. Transgene persistence in transduced terminal postmitotic cells is long-lasting, on the span of years [24–26]. We previously observed that AAV vector-mediated systemic delivery of the GDF11 propeptide-Fc (GDF11PRO-Fc) transgene into neonatal mice augmented skeletal muscle growth [14]. From these findings, we sought to further investigate the impact of both local and systemic GDF11PRO-Fc expression on skeletal muscle mass and function in adult mice and determine whether these effects have therapeutic relevance in the mdx mouse model of Duchenne muscular dystrophy (DMD). In the present study, we find that GDF11PRO-Fc induces skeletal muscle hypertrophy after gene delivery in adult mice. Furthermore, we observe that systemic GDF11PRO-Fc gene delivery improves muscle strength and reduces intramuscular fibrosis in mdx mice; however, GDF11PRO-Fc does not improve the dystrophic pathology overall.

## Methods

### Pull-down assay

To obtain GDF11PRO-Fc and myostatin propeptide-Fc (MPRO-Fc) fractions, HEK293 cells were transiently transfected by calcium phosphate precipitation with a plasmid containing the sequence for GDF11PRO-Fc or MPRO-Fc. Forty-eight hours after transfection, cell lysates containing GDF11PRO-Fc or MPRO-Fc protein were collected with a mild lysis buffer (0.025 M Tris, 0.15 M NaCl, 0.001 M EDTA, 1% NP-40, 5%, glycerol, pH 7.4). To assess complex formation, rGDF11 (1958-GD-010; R&D Systems; Minneapolis, MN), recombinant MSTN (rMSTN; 788-G8–010; R&D Systems; Minneapolis, MN) or recombinant activin A (rActivinA; 338-AC-010; R&D Systems; Minneapolis, MN) were added to cell lysates to a final concentration of 100–500 ng/ml. Pull-down assay was performed using the Pierce Classic IP Kit (Thermo Scientific; Waltham, MA) according to the manufacturer’s instructions. Briefly, combined lysate fractions containing GDF11PRO-Fc or MPRO-Fc and rGDF11, rMSTN, or rActivin A were incubated with protein A/G agarose resin with end-over-end mixing for 1 h at 4 °C. The resins were washed to remove unbound proteins, and protein A/G-Fc complexes were eluted with 2X SDS-PAGE reducing sample buffer (0.3 M Tris-HCl, 20 mM DTT, 5% SDS, 50% glycerol; pH 6.8) at 100 °C for 5 min. Fractions were then separated on a 12% SDS-PAGE gel, transferred to a 0.22 μm PVDF membrane, and probed for GDF11PRO-Fc or MPRO-Fc and rGDF11, rMSTN, or rActivin A. The antibodies used were goat anti-human IgG Fc-specific (1:5000; I2136; Millipore Sigma; St. Louis, MO), mouse anti-GDF11 (1:1000; MAB19581; R&D Systems; Minneapolis, MN), goat anti-MSTN (1:1000; AF788; R&D Systems; Minneapolis, MN), and goat anti-activin A beta A subunit (1:1000; AF338; R&D Systems; Minneapolis, MN).

### AAV vector production

Construction of the GDF11 and GDF11PRO-Fc transgene cassettes for AAV packaging has been previously described (Additional file 1: Figure S1 and S2) [14]. Briefly, the codon-optimized sequences for human GDF11 and human GDF11PRO-Fc were synthesized (GenScript; Piscataway, NJ) and subcloned into an AAV expression vector with a CAG (cytomegalovirus [CMV] early enhancer, first exon and first intron of chicken β-actin gene, and splice acceptor of rabbit β-globin) promoter for ubiquitous expression (Additional file 1: Figure S2). The AAV expression vector contains the inverted terminal repeat (ITR) sequences necessary for packaging into AAV. For generation of the GDF11PRO-Fc D122A construct, the D122A point mutation was added via site-directed mutagenesis using the GDF11PRO-Fc sequence as a template [21]. The primers used were 5′-GCCGCTCTGCAGCCCGAGGACTTCCTG (forward) and 5′-GCCCTGGAAATCGTGCAGGTCCAGGATCTGC (reverse). AAV6 and AAV9 vectors were generated using the triple-plasmid transfection method in HEK293 cells [27]. Forty-eight to seventy-two hours post-transfection, cells and media were collected and vectors were purified by polyethylene glycol (PEG) precipitation followed by two rounds of cesium chloride (CsCl) density ultracentrifugation. AAV titers were determined by DNA dot blot and vectors were stored at − 80 °C.

### C2C12 culture and analysis

C2C12 myoblasts were seeded on 12-well plates and cultured in proliferation media (DMEM + 10% FBS, 1% penicillin/streptomycin [P/S]). Upon reaching 70% confluency, media was changed to differentiation media (DMEM + 2% horse serum, 1% P/S) to induce differentiation. Differentiation media was changed at least every 2 days. For post-differentiation studies, C2C12 myoblasts were differentiated into myotubes for 5 days prior to infection with AAV6-EGFP or AAV6-GDF11PRO-Fc at a multiplicity of infection (MOI) of 10 [5]. Forty-eight hours after viral vector treatment, 100 ng/ml rGDF11 or rMSTN was added. For pSMAD2/3 and SMAD2/3 immunoblotting, cells were lysed and analyzed 24 h after the addition of rGDF11 or rMSTN. For analysis of myotube diameter and nuclear content, myotubes were stained and analyzed 72 h after addition of rGDF11/rMSTN using a rabbit anti-dystrophin antibody (R22/R23; Xiao Xiao lab). DAPI was used to stain myonuclei. Myotubes were analyzed if they stained positively for dystrophin, had a defined cytoplasmic compartment fully within the frame, and contained 2 or more myonuclei. Myotube diameters were measured at three distinct points and the average was used for calculations. The differentiation index was defined as the percentage of myonuclei contained within myotubes relative to the total number of myonuclei. Experiments in C2C12 cells were repeated 3 times.

### Intracellular vector genome quantification

Prior to DNA collection, C2C12 cultures were washed with PBS and briefly treated with trypsin to selectively detach myotubes. Harvested myotubes were centrifuged at 500×*g* for 5 min and the cell pellet was washed 3 times with PBS. Quantification of vector genomes was performed by extracting total DNA using the DNeasy Blood & Tissue Kit (Qiagen; Hilden, Germany), according to the manufacturer’s instructions. Total vector genome copy number was obtained by qPCR using TaqMan probes recognizing the CMV promoter sequence on an Applied Biosystems 7300 Real-Time PCR System (ThermoFisher; Waltham, MA). Values were normalized to endogenous mouse glucagon to obtain the vector genome copy number per diploid genome. Absolute copy numbers were calculated based on a standard curve generated from serial dilutions of pENTR11-AAV-D(+)-EGFP, pXX-CAG-GDF11PRO-Fc, and pBSKS-mouse-glucagon. Primer and probe sequences used were CMV forward primer: 5′-GTATGTTCCCATAGTAACGC, CMV reverse primer: 5′- GGCGGACTTGGCATATGATACACT, CMV probe: 5′-FAM-TCAATGGGTGGAGTATTTA, mouse glucagon forward primer: 5′-AAGGGACCTTTACCAGTGATGTG, mouse glucagon reverse primer: 5′-ACTTACTCTCGCCTTCCTCGG, mouse glucagon probe: 5′-FAM-CAGCAAAGGAATTCA.

### Mice and vector administration

C57BL/6J, C57BL/10J, and C57BL/10ScSn-DMD^mdx^/J (mdx) mice were purchased from the Jackson Laboratory (Bar Harbor, ME). Mice were maintained in a 12-h:12-h light:dark artificial light cycle (0700–1900 h) at a temperature of 20 °C and a humidity of 55 ± 5%. For intramuscular vector administration experiments, 8-week-old male C57BL/6J mice were randomized into treatment or control groups and treated with AAV9-GDF11PRO-Fc (*n* = 5) or vehicle (*n* = 5) at a total dose of 1 × 10^12^ vg/kg (~ 2.5 × 10^10^ vg/mouse) into the right-side gastrocnemius and tibialis anterior. For trials in dystrophic mice, 6-week-old male mdx mice were randomized into treatment or control groups and injected with AAV9-GDF11PRO-Fc (*n* = 7), AAV9GDF11PRO-Fc D122A (*n* = 7) or vehicle (*n* = 7) at a dose of 3 × 10^13^ vg/kg (~ 1 × 10^12^ vg/mouse) via tail vein injection. Vehicle-injected male 6-week-old C57BL/10J mice (*n* = 5) were used as a wild type control. All animals had ad libitum access to food and water. For non-survival surgery, mice were anesthetized with 2.5% 2,2,2-tribromoethanol (Avertin). Blood for serum analysis was obtained from the retro-orbital sinus. The entire tibialis anterior, gastrocnemius, quadriceps, diaphragm, and heart were surgically removed, snap frozen in 2-methylbutane, and stored at − 80 °C.

### Muscle functional tests

Measurement of forelimb/hindlimb grip strength was conducted using a grip force meter (Chatillon DFE2–002; AMETEK; Largo, FL, USA) to measure peak grip strength. The mouse was moved toward the grip bar and positioned so that the bar was grasped with one or both paws. Then, the mouse was slowly pulled back until the mouse could no longer hold on to the bar. The peak strength was recorded. For each mouse, a total of 5 measurements at 1-min intervals were obtained. The highest and lowest grip strength reading was omitted, and the remaining 3 measurements were averaged. For the measurement of running endurance, the treadmill apparatus (Harvard Apparatus; Holliston, MA) was positioned on a flat surface with shock pads set to 1 mA. The mice were placed on the treadmill and the starting speed was set to 5 m/min for 1 min. The speed was then increased to 10 m/min for 5 min and continually increased in 5 m/min increments every 5 min. A mouse was removed from the apparatus when it failed to re-enter the treadmill after 7 s. The total distance traveled was recorded for analysis. For the rotarod test (ENV-574 M; Med Associates; St. Albans, VT), mice were placed on the stationary rod and allowed to acclimate for 30 s. Then, the rotational speed was adjusted to the load rpm for 30 s. Any mice that fell during the load period were placed back on the rod. After the load period, the rotarod was gradually accelerated from 3.0 to 30 rpm. The time to fall from this point was recorded for analysis. Mice were tested a total of 3 times per session and the highest recorded time before falling off the rotarod was used for analysis. For all functional tests, mice were trained and acclimated to equipment for at least two sessions prior to data collection. All functional tests were conducted by a single experimenter blinded to the experimental groups.

### SDS-PAGE and western blot

C2C12 myotubes were lysed in RIPA buffer (25 mM Tris-HCl, 150 mM NaCl, 1% Triton X-100, 0.5% sodium deoxycholate, 0.1% SDS, pH 7.4) and 20 μg total protein was loaded per lane. Snap-frozen mouse liver and muscle tissue samples were homogenized in T-PER Tissue Protein Extraction Reagent (Thermo Fisher; Waltham, MA) and 50 μg total protein was loaded per lane. All lysates were supplemented with protease and phosphatase inhibitor cocktail (Millipore Sigma; St. Louis, MO). Protein concentration was determined by Pierce BCA Protein Assay Kit (ThermoFisher; Waltham, MA). For western blot of serum proteins, 0.5 μl of serum was diluted in PBS. Samples were reduced in Laemmli sample buffer (62.5 mM Tris-HCl, 1.5% SDS, 8.3% glycerol, 1.5% β-mercaptoethanol, 0.005% bromophenol blue, pH 6.8) by boiling for 5 min. Samples were separated at 80–120 V on a 10–12% SDS-PAGE gel and transferred to a 0.2-μm polyvinylidene fluoride (PVDF) membrane. Membranes were washed in 0.1% TBS-T (10 mM Tris-HCl, 100 mM NaCl, 0.1% Tween-20, pH 7.5). Five percent non-fat dry milk in 0.1% TBS-T was used to block the membrane for 1 h at room temperature. For phosphoprotein detection, an alternative blocking buffer (5% bovine serum albumin [BSA] in 0.1% TBS-T) was used. For protein detection, membranes were incubated with primary antibodies overnight at 4 °C and horseradish peroxidase (HRP)-conjugated secondary antibodies for 1 h at room temperature. Protein bands were detected with Western Lightning ECL Pro (PerkinElmer; Waltham, MA). Equal protein loading was confirmed by Ponceau S staining. For cell and tissue lysates, GAPDH was used as a loading control. For stripping, PVDF membranes were incubated for 5–15 min in mild stripping buffer (Restore Western Blot Stripping Buffer; Thermo Fisher; Waltham, MA) at room temperature. The primary antibodies used were rabbit anti-SMAD2/3 (1:1000; 3102S; Cell Signaling; Danvers, MA), rabbit anti-pSMAD2/3 (Ser 423/425; 1:1000; sc-11,769-R; Santa Cruz Biotechnology; Dallas, TX), goat anti-human IgG (Fc-specific; 1:1000; I2136; Sigma Aldrich; St. Louis, MO), and rabbit anti-GAPDH (1:5000; G9545; Sigma Aldrich; St. Louis, MO).

### Immunofluorescence staining and analysis

Ten-micrometer gastrocnemius cryosections were cut from the midbelly of the muscle bundle. For myofiber analysis, sections were stained with AlexaFluor-488-conjugated wheat germ agglutinin (WGA; 1:50; Thermo Fisher; Waltham, MA). For analysis of myofiber minimum Feret diameter (MFD) and cross-section area (CSA), at least 500 individual myofibers per mouse were measured from each quadrant of the muscle cross-section. For the determination of the proportion of myofibers exhibiting central nucleation, the number of myofibers with only peripheral nuclei was subtracted from the total number of myofibers and the difference was divided by the total number of myofibers. Intramuscular collagen deposition was assessed by Masson trichrome staining (MTC; IMEB; San Marcos, CA). For calculation of the fibrotic area percentage, the total fibrotic area was divided by the total area of the entire muscle cross-section. For determination of membrane IgG permeability, muscle cross-sections were fixed for 5 min on ice with a 1:1 solution of acetone/methanol and co-stained with AlexaFluor-488-conjugated WGA (1:50) and Cy3-conjugated goat anti-mouse IgG (1:50; AP124C; Sigma Aldrich; St. Louis, MO) for 2 h at room temperature in the dark. For assessment of membrane IgG permeability, the total area of IgG positive myofibers was divided by the entire muscle cross-section area. All image analyses were conducted in ImageJ/Fiji [28, 29].

### Creatine kinase assay

Serum creatine kinase (CK) was quantified using a kit (Pointe Scientific; Canton, MI) according to the manufacturer’s instructions. Briefly, the transphosphorylation of ADP to ATP by CK was measured by measuring the increase in NADPH absorbance at 340 nm [30]. The change in absorbance at 340 nm per min was used to calculate the serum CK level.

### Statistical analysis

Values are presented as mean ± SEM. Student’s *t* test was used to compare two groups. One-way ANOVA was used to compare three or more groups. All statistical analyses were conducted in GraphPad Prism (GraphPad Software; La Jolla, CA). *p* < 0.05 was considered statistically significant.

## Results

### GDF11PRO-Fc binds to both GDF11 and MSTN

**Fig. 1:**
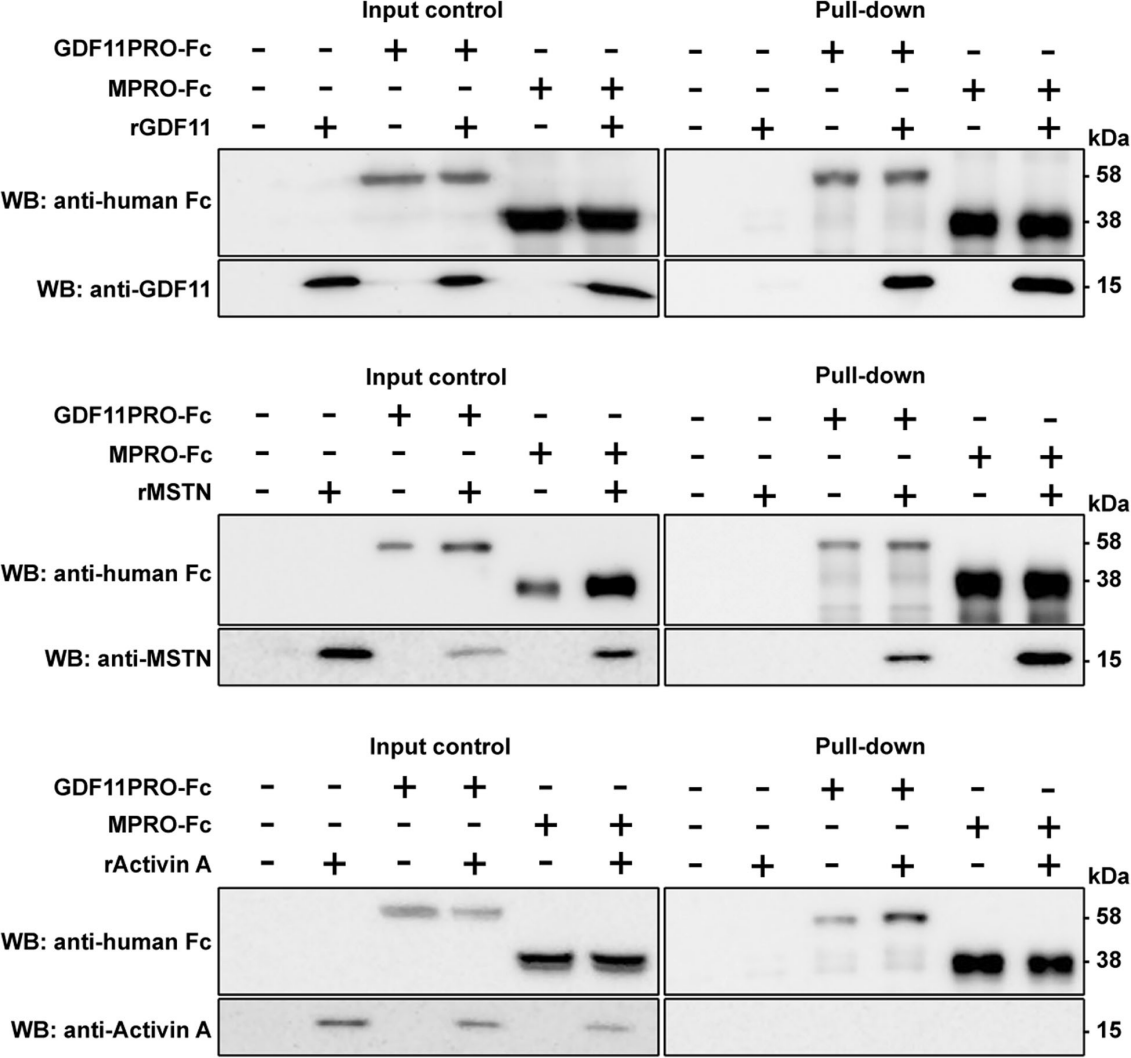
GDF11PRO-Fc associates with GDF11 and MSTN. Protein-protein interactions between GDF11PRO-Fc or MPRO-Fc and rGDF11, rMSTN, or rActivin A were determined by a pull-down assay. GDF11PRO-Fc or MPRO-Fc was incubated with rGDF11, rMSTN, or rActivin A for 1 h at 4 °C. Fc-fused protein complexes were separated on a protein A/G-coated agarose resin and eluates were run on a 12% SDS-PAGE gel under reducing conditions and probed by western blot. Input control was 5% of the input material. WB western blot

To show that GDF11PRO-Fc can sequester the active mature GDF11 dimer via a direct interaction, we aimed to identify a protein-protein interaction between GDF11 and GDF11PRO-Fc. Additionally, due to the close structural homology of the GDF11 and MSTN mature dimers, we also wanted to determine whether GDF11PRO-Fc associates with MSTN. To identify these protein-protein interactions, we performed a set of pull-down assays (Fig. 1). The ligands rGDF11, rMSTN, and rActivin A were incubated with lysate fractions from HEK293 cells transfected with a plasmid encoding for GDF11PRO-Fc or MPRO-Fc at pH 7.4. Owing to the conjugated Fc fragments on the modified propeptides, fractions could be pulled down directly on a protein A/G agarose resin. As expected, GDF11PRO-Fc effectively pulled down both rGDF11 and rMSTN, indicating that GDF11PRO-Fc was capable of binding both ligands. In addition, MPRO-Fc successfully pulled down both rGDF11 and rMSTN as well. Both GDF11PRO-Fc and MPRO-Fc were not able to pull down the more distantly related TGF-β superfamily ligand rActivin A, indicating that ligand binding is specific (Fig. 1). No binding of GDF11PRO-Fc, MPRO-Fc, rGDF11, rMSTN, or rActivin A were observed on a control agarose resin without protein A/G. From these results, we conclude that GDF11PRO-Fc spontaneously associates with the mature GDF11 and MSTN dimers at physiological pH.

### GDF11PRO-Fc blocks GDF11/MSTN-induced atrophy in C2C12 myotubes

**Fig. 2:**
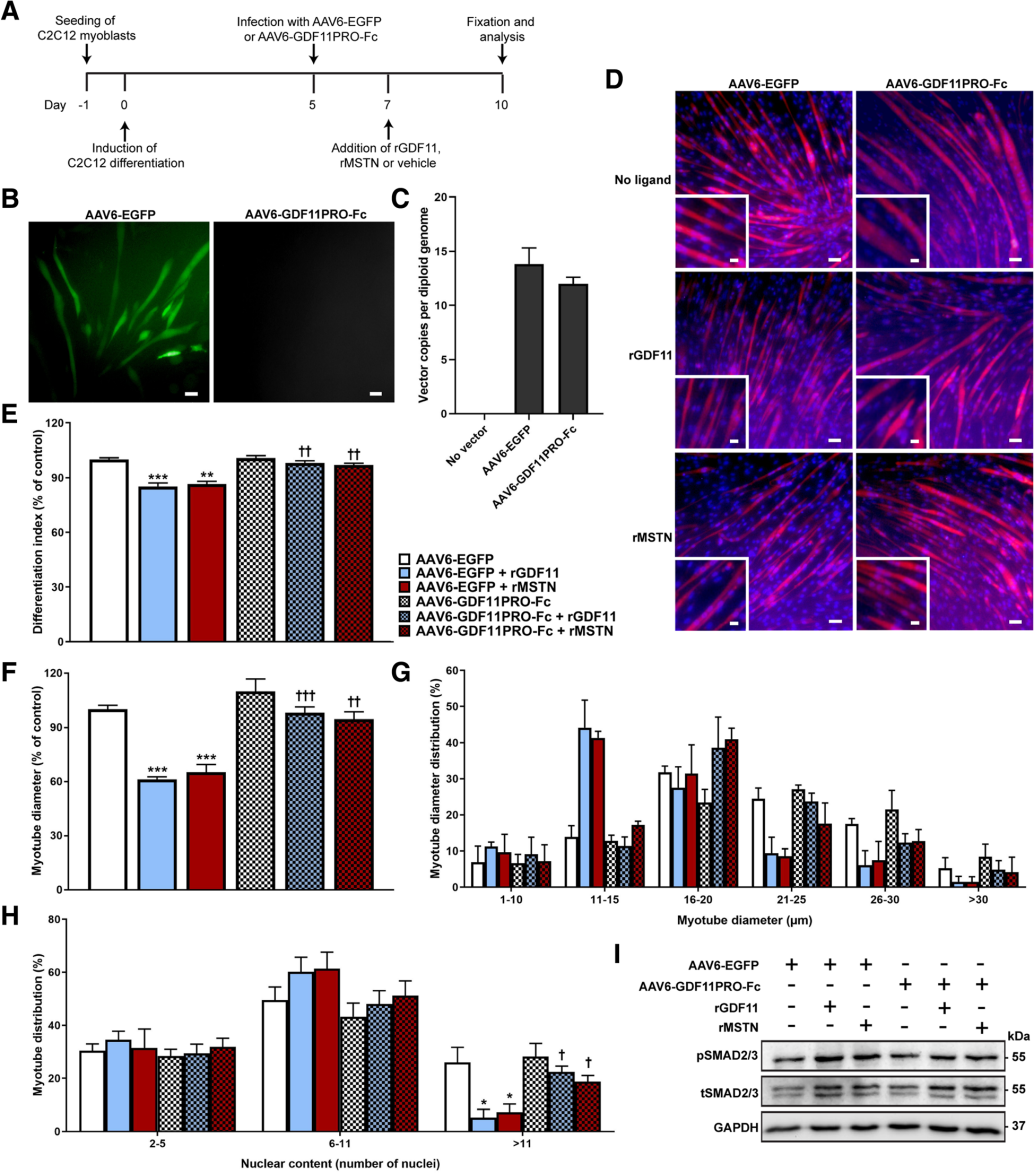
GDF11PRO-Fc blocks GDF11/MSTN-induced myotube atrophy in C2C12 cells. **a** Schematic detailing experimental timeline in C2C12 myotubes. AAV6-EGFP or AAV6-GDF11PRO-Fc was added to C2C12 myotubes at a MOI of 10^5^ on day 5 post-differentiation and 100 ng/ml rGDF11 or rMSTN was added on day 7. Myotubes were stained and analyzed on day 10. **b** EGFP expression was evident at 48–72 h in C2C12 myotubes treated with AAV6-EGFP (MOI 10 [5]). Scale bars represents 50 μm. **c** Vector genome copy number per diploid genome in C2C12 myotubes 72 h after addition of AAV6-EGFP or AAV6-GDF11PRO-Fc (MOI 10 [5]). **d** Representative immunofluorescence images of C2C12 myotubes. C2C12 myotube membranes were visualized by staining with an anti-dystrophin antibody (red). Nuclei were stained with DAPI (blue). Inset shows a zoomed-in region. Scale bars represent 50 μm (main panel) and 25 μm (panel inset). **e** The fraction of nuclei incorporated into myotubes (differentiation index) was calculated and presented as a percentage of control. **f** Average myotube diameter relative to control and (**g**) distribution of diameter measurements. For myotube diameter measurements, each myotube was measured at three points along the length of the myotube and averaged. **h** Number of nuclei incorporated per myotube. A minimum of 50 myotubes were analyzed per experimental condition. **i** pSMAD2/3 relative to tSMAD2/3 was assessed by western blot. Equal protein loading was verified by Ponceau S staining and GAPDH was used as a loading control. Data represents results from three separate experiments. All error bars represent mean ± SEM. *p < 0.05; **p < 0.01; ***p < 0.001; n.s. not significant; compared to AAV6-EGFP-treated control. †*p* < 0.05; ††*p* < 0.01; †††*p* < 0.001; compared to AAV6-EGFP + ligand-treated. pSMAD2/3: phosphorylated SMAD2/3; tSMAD2/3: total SMAD2/3

Addition of rGDF11 and rMSTN to differentiated myotubes has previously been shown to induce atrophy in murine C2C12 and primary human skeletal myotubes [16, 31]. Having determined that GDF11PRO-Fc binds GDF11 and MSTN, we next asked if GDF11PRO-Fc could prevent GDF11/MSTN-induced myotube atrophy in differentiated C2C12 myotubes. To achieve this, we packaged the DNA sequence encoding for GDF11PRO-Fc into the AAV6 capsid to generate the AAV6-GDF11PRO-Fc vector. In these experiments, the AAV6 vector was selected for its ability to efficiently and selectively transduce differentiated C2C12 myotubes, but not myoblasts [32, 33]. C2C12 myoblasts were cultured and differentiated for 5 days prior to treatment with AAV6-GDF11PRO-Fc or AAV6-EGFP (control vector) at a MOI of 10^5^ (Fig. 2a). As expected, GFP expression was observable in C2C12 myotubes infected with AAV6-EGFP at 48–72 h post-infection, and quantification of internalized vector genome copies per diploid genome at 72 h post-infection indicated that vector transduction was adequately achieved (Fig. 2b and c).

 Forty-eight hours after AAV treatment, rGDF11 or rMSTN was added to the media to a final concentration of 100 ng/ml. Seventy-two hours later, myotubes were fixed and stained for immunofluorescence analysis using an antibody recognizing dystrophin (rod 22/rod 23) to visualize myotube peripheral membranes and DAPI to stain myonuclei (Fig. 2d). A reduction in the differentiation index, which is defined as the proportion of myonuclei incorporated into myotubes, was observed in myotubes treated with rGDF11 (− 15%, *p* = 0.0008) or rMSTN (− 14%, *p* = 0.0013) relative to untreated control. However, this effect was blunted in GDF11PRO-Fc-expressing C2C12 myotubes treated with rGDF11 (− 1.9%; *p* = 0.0047, compared to rGDF11-treated control) or rMSTN (− 3.0%; *p* = 0.0040, compared to rMSTN-treated control; Fig. 2e). In addition, C2C12 myotubes that were treated with AAV6-GDF11PRO-Fc resisted rGDF11 or rMSTN-induced myotube atrophy. A substantial decrease in the average myotube diameter relative to control was detected in myotubes treated with rGDF11 (− 39%; 0.0002) or rMSTN (− 35%; *p* = 0.0003) compared to control. On the contrary, myotubes expressing GDF11PRO-Fc treated with rGDF11 (− 1.8%; *p* = 0.0005, compared to rGDF11-treated control) or rMSTN (− 5.3%; *p* = 0.0075, compared to rMSTN-treated control) were largely unaffected (Fig. 2f and g). In a separate experiment, GDF11PRO-Fc was not able to prevent rActivin A-induced myotube atrophy, which is in line with the observation that GDF11PRO-Fc does not bind activin A (Additional file 1: Figure S3).

Treatment with rGDF11 or rMSTN was also associated with a lower proportion of mature myotubes with higher numbers of myonuclei, which suggests an inhibition of myotube differentiation. Myotubes with more than 11 myonuclei comprised only 5.2% (*p* = 0.0215) and 7.2% (*p* = 0.0328) of myotubes analyzed in cells treated with rGDF11 and rMSTN, respectively. In comparison, myotubes with more than 11 nuclei made up 26% of myotubes analyzed in control myotubes. In myotubes expressing GDF11PRO-Fc that were treated with rGDF11 or rMSTN, the proportion of myotubes with more than 11 myonuclei was 22% (*p* = 0.0104, compared to rGDF11-treated control) and 19% (*p* = 0.0404, compared to rMSTN-treated control), respectively (Fig. 2h). Finally, in agreement with what would be expected following GDF11/MSTN signaling at ActRII, an increase in phosphorylated SMAD2/3 (pSMAD2/3) protein levels in myotubes treated with AAV6-EGFP and rGDF11 or rMSTN was observable on western blot 24 h after addition of ligand (Fig. 2i). This increase in pSMAD2/3 levels was absent in myotubes treated with AAV6-GDF11PRO-Fc, suggesting that GDF11PRO-Fc expression antagonizes GDF11/MSTN-induced activation of SMAD2/3 in differentiated myotubes. Overall, these results indicate that GDF11PRO-Fc was able to prevent rGDF11 and rMSTN-induced myotube atrophy and inhibition of differentiation in C2C12 cells.

### Localized GDF11PRO-Fc expression induces skeletal muscle hypertrophy in adult mice

Previously, we have demonstrated that systemic AAV vector-mediated gene delivery of GDF11PRO-Fc into neonatal mice led to a significant increase in the rate of skeletal muscle growth [14]. However, it was unclear from this study whether or not GDF11PRO-Fc simply enhanced skeletal muscle growth or actually induced skeletal muscle hypertrophy. Furthermore, it was not possible to determine if the effects of GDF11PRO-Fc were mediated by local blockade of GDF11/MSTN in skeletal muscle or if the observed effects were due to systemic modulation of GDF11/MSTN. To address these questions, we evaluated the impact of intramuscular AAV9-GDF11PRO-Fc administration in adult C57BL/6J mice. In these studies, only the right-side hindlimb was injected, and the contralateral left-side hindlimb was used as a control.

**Fig. 3:**
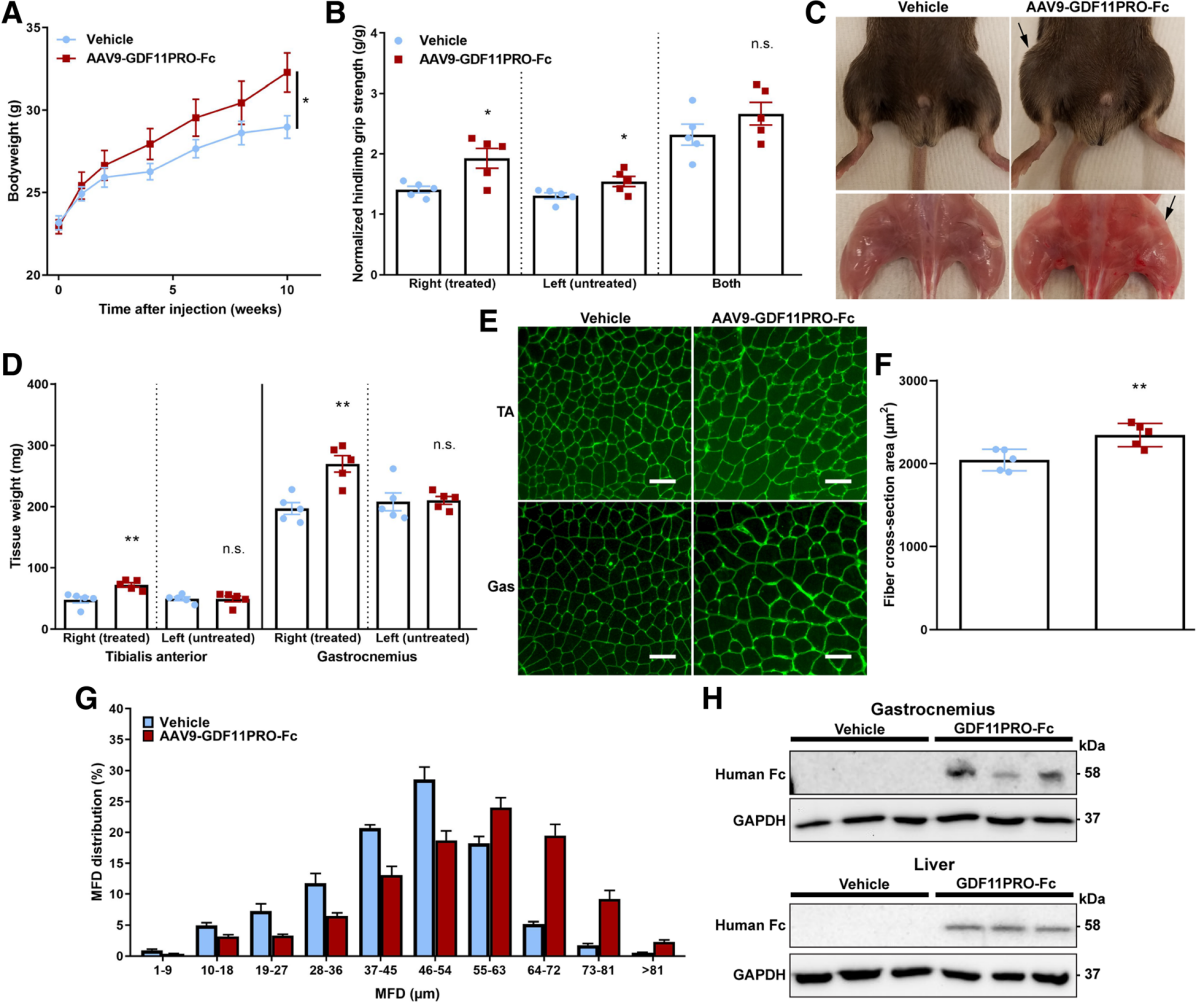
GDF11PRO-Fc induces localized skeletal muscle hypertrophy after intramuscular gene delivery. 8-week-old C57BL/6J mice were treated with AAV9-GDF11PRO-Fc (*n* = 5) or vehicle (*n* = 5) via unilateral intramuscular injection into the right-side hindlimb. The contralateral left-side hindlimb was not treated. Mice were euthanized 10 weeks post-treatment. **a** Average bodyweight over time. **b** Hindlimb grip strength normalized to bodyweight at 10 weeks post-treatment. Shown are measurements from a single hindlimb and both hindlimbs. **c** Representative gross hindlimb musculature. The injected hindlimb is designated with a black arrow. **d** Wet tissue weight of tibialis anterior and gastrocnemius. **e** Representative immunofluorescence images of injected right-side gastrocnemius cross-sections stained with AlexaFluor-488-conjugated WGA to visualize myofibers. Scale bars represent 100 μm. **f** Average myofiber cross-section area in the injected right-side gastrocnemius. **g** Myofiber MFD distribution in the injected right-side gastrocnemius. A minimum of 500 myofibers were measured per mouse. **h** Western blot identification of GDF11PRO-Fc in tissue lysates from injected right-side gastrocnemius and liver samples of treated mice. GDF11PRO-Fc was not detectable in vehicle-treated mice. Equal protein loading was verified by Ponceau S staining and GAPDH was used as a loading control. All error bars represent mean ± SEM. *p < 0.05; **p < 0.01; n.s. not significant; compared to vehicle-treated control. TA tibialis anterior, Gas gastrocnemius

Bodyweight increased slightly over time in mice treated with AAV9-GDF11PRO-Fc (+ 10% at 10 weeks; *p* = 0.0418; Fig. 3a). In addition, single-hindlimb grip strength normalized to body weight was significantly increased in both limbs tested individually, with the greater magnitude of effect seen in the treated right-side hindlimb (+ 37%; *p* = 0.0177), although a significant increase in normalized grip strength in the untreated left-side hindlimb (+ 18%; *p* = 0.0426) was also observed. However, this difference did not reach significance when both hindlimbs were tested simultaneously (+ 15%; *p* = 0.2375; Fig. 3b). Ten weeks after vector administration, the right-side hindlimb was visibly larger in mice treated with AAV9-GDF11PRO-Fc (Fig. 3c). In mice treated with AAV9-GDF11PRO-Fc, the wet tissue mass of the injected right-side tibialis anterior (+ 50%; *p* = 0.0046) and gastrocnemius (+ 37%; *p* = 0.0023) was significantly higher in comparison to vehicle-treated controls. There was no statistically significant difference in the wet tissue mass of the untreated left-side hindlimb muscles (Fig. 3d). Myofiber area analysis revealed an increase in the average myofiber cross-section area (+ 15%; *p* = 0.0078) in the AAV9-GDF11PRO-Fc-injected right-side gastrocnemius (Fig. 3e and f). MFD distribution analysis also revealed a shift toward larger myofibers in the right-side gastrocnemius of mice injected with AAV9-GDF11PRO-Fc (Fig. 3g), indicating that localized GDF11PRO-Fc expression induced muscle hypertrophy. In a separate cohort, we also assessed the impact of localized gene delivery of GDF11 by an intramuscular injection of AAV9-GDF11 into the right-side hindlimb, and significant muscle atrophy was observed 10 weeks post-treatment in the injected hindlimb (Additional file 1: Figure S4).

Western blot analysis indicated that the GDF11PRO-Fc protein was expressed in the AAV9-GDF11PRO-Fc-injected right-side gastrocnemius (58 kDa band; Fig. 3h). However, GDF11PRO-Fc was not detectable in the untreated left-side gastrocnemius at the same total protein amount loaded, suggesting that the majority of skeletal muscle GDF11PRO-Fc expression was localized to the injected right-side hindlimb (data not shown). GDF11PRO-Fc was also detectable in the liver by western blot indicating that some systemic exposure had occurred (Fig. 3h). This observation can most likely be attributed to vascular leakage of the AAV9 vector into systemic circulation from the site of injection [34]. From these data, we determine that GDF11PRO-Fc induces skeletal muscle hypertrophy in adult mice. Additionally, these effects are at least partially mediated by local blockade of GDF11/MSTN on skeletal muscle, probably via inhibition of ligand interactions with ActRII on skeletal muscle tissue.

### Systemic GDF11PRO-Fc gene delivery increases skeletal muscle mass and strength in mdx mice

Next, we evaluated the impact of systemic GDF11PRO-Fc expression in mdx mice to determine if GDF11PRO-Fc could also induce hypertrophy and increase strength in dystrophic skeletal muscle. For the purposes of this trial, the GDF11PRO-Fc construct was modified with a mutation to render it impervious to endogenous BMP1/TLD-like metalloproteinase cleavage (GDF11PRO-Fc D122A) and increase factor persistence in systemic circulation [21]. For these experiments, both AAV9-GDF11PRO-Fc and AAV9-GDF11PRO-Fc D122A were evaluated to establish whether the mutated D122A transgene would lead to a more pronounced effect in vivo. To achieve systemic expression, mdx mice were treated with vector or vehicle by tail vein injection. Following intravenous delivery, the AAV9 vector transduces the mouse liver with high efficiency. Transduced hepatocytes can then express the transgene and secrete the protein product into systemic circulation [35].

**Fig. 4:**
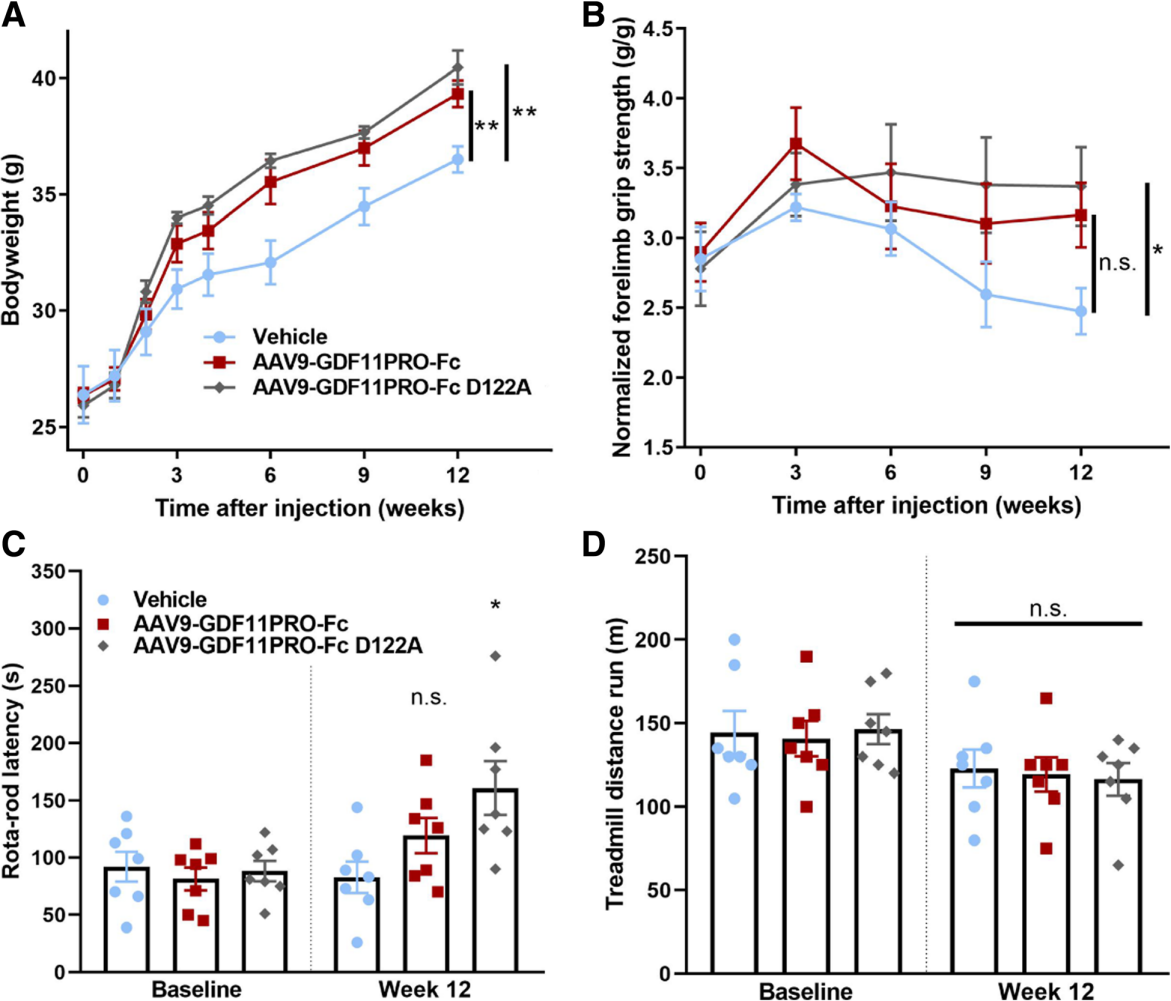
GDF11PRO-Fc improves grip strength and rotarod performance in dystrophic mdx mice. 6-week-old mdx mice were treated with AAV9-GDF11PRO-Fc (*n* = 7), AAV9-GDF11PRO-Fc D122A (*n* = 7), or vehicle (*n* = 7) via tail vein injection. **a** Average bodyweight over time. **b** Forelimb grip strength normalized to bodyweight over time. **c** Best rotarod time-to-fall recorded from pre-treatment (baseline) and 12 weeks post-treatment. The best time from three attempts was used for analysis. **d** Total distance run on treadmill endurance test from pre-treatment (baseline) and 12 weeks post-treatment. All error bars represent mean ± SEM. *p < 0.05; **p < 0.01; n.s. not significant; compared to vehicle-treated control

Starting from 3 weeks post-injection, we observed a significant increase in body weight that persisted for the duration of the trial with AAV9-GDF11PRO-Fc (+ 7.7% at 12 weeks; *p* = 0.0094) and AAV9-GDF11PRO-Fc D122A (+ 11% at 12 weeks; *p* = 0.006) treatment (Fig. 4a). It should be noted that, due to the dystrophic pathology, mdx mice typically exhibit compensatory muscle hypertrophy, which may partially mask the increase in muscle mass induced by GDF11PRO-Fc expression. In regards to muscle function tests, treated mice displayed increased forelimb grip strength, even after normalizing to bodyweight. However, the difference in normalized forelimb grip strength only reached statistical significance in the AAV9-GDF11PRO-Fc D122A group. At 12 weeks post-treatment, the average normalized forelimb grip strength was increased by + 28% (*p* = 0.0873) and + 36% (*p* = 0.0248) in mice treated with AAV9-GDF11PRO-Fc and AAV9-GDF11PRO-Fc D122A, respectively (Fig. 4b). Rotarod performance was also improved by AAV9-GDF11PRO-Fc D122A treatment on average (+ 93% increase in rotarod latency time; *p* = 0.0127). Rotarod performance was also improved in the AAV9-GDF11PRO-Fc group, but this difference did not reach statistical significance (+ 44% increase in rotarod latency time; *p* = 0.2835; Fig. 4c). There was no difference observed in treadmill running time across any of the groups (Fig. 4d).

**Fig. 5:**
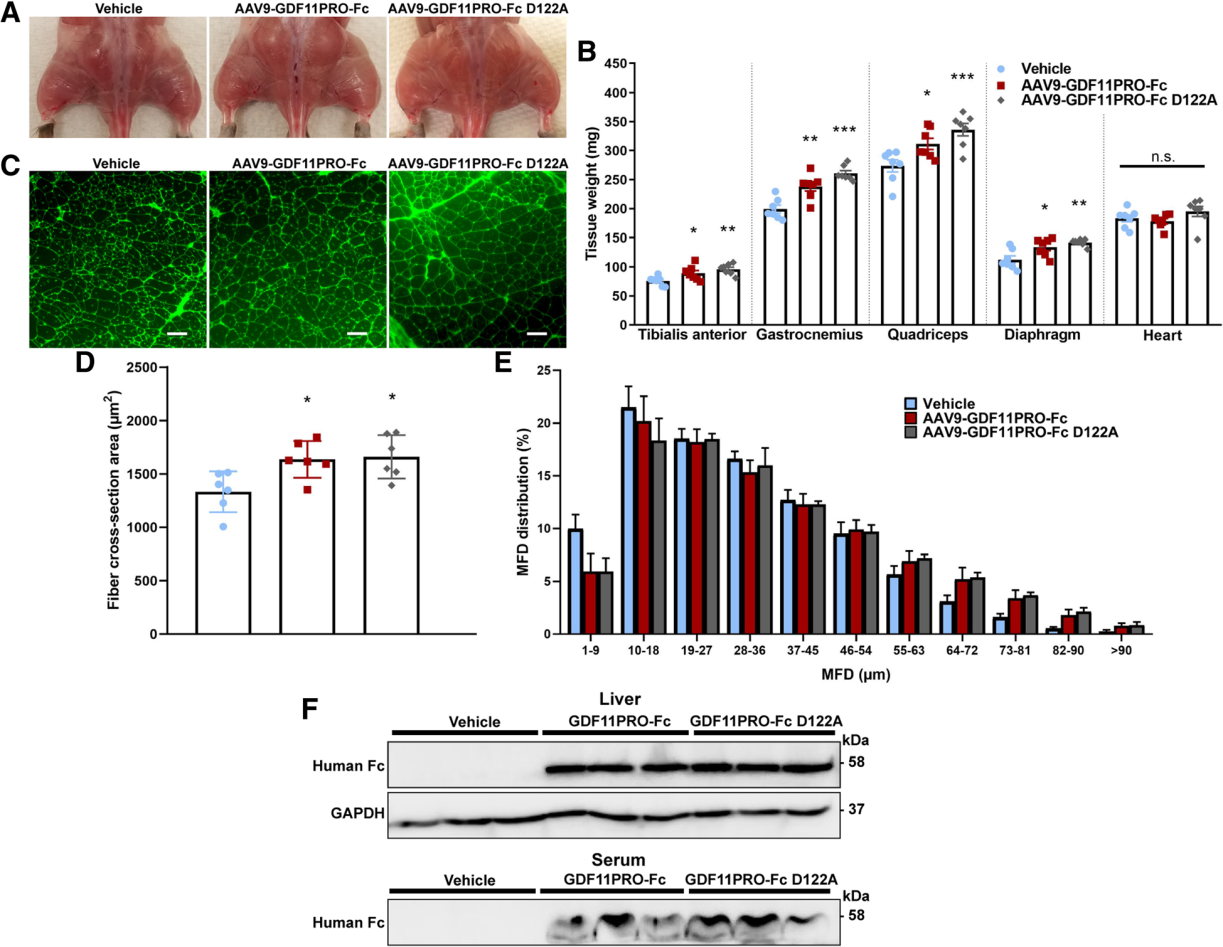
GDF11PRO-Fc induces skeletal muscle hypertrophy in dystrophic mdx mice. 6-week-old mdx mice were treated with AAV9-GDF11PRO-Fc (*n* = 7), AAV9-GDF11PRO-Fc D122A (*n* = 7), or vehicle (*n* = 7) via tail vein injection. Mice were euthanized 12 weeks post-treatment. **a** Representative gross hindlimb musculature. **b** Wet tissue weight of limb muscles, diaphragm, and heart. **c** Representative immunofluorescence images of gastrocnemius cross-sections stained with AlexaFluor-488-conjugated WGA to visualize myofibers. Scale bars represent 100 μm. **d** Average myofiber cross-section area in the gastrocnemius. **e** Myofiber MFD distribution in the gastrocnemius. A minimum of 500 myofibers were measured per mouse. **f** Identification of GDF11PRO-Fc by western blot in liver tissue lysate and serum of mice treated with AAV9-GDF11PRO-Fc. Equal protein loading was verified by Ponceau S staining and GAPDH was used as a loading control in liver tissue lysates. *p < 0.05; **p < 0.01; ***p < 0.001; n.s. not significant; compared to vehicle-treated control

At the end of the 12-week trial, muscle hypertrophy was grossly evident in the treated mice (Fig. 5a). Average wet tissue masses of the tibialis anterior, gastrocnemius, and quadriceps were increased by + 17% (*p* = 0.0472), + 20% (*p* = 0.0011), and + 14% (*p* = 0.0333), respectively, in the AAV9-GDF11PRO-Fc group. In the AAV9-GDF11PRO-Fc D122A group, these values were further increased to + 26% (*p* = 0.0030), + 31% (*p* = 0.0003), and + 23% (*p* = 0.0009) over vehicle-treated control for the change in average tibialis anterior mass, gastrocnemius mass, and quadriceps mass, respectively. In addition, the diaphragm wet tissue mass was increased by + 19% (*p* = 0.0162) and + 26% (*p* = 0.0014) in the AAV9-GDF11PRO-Fc and AAV9-GDF11PRO-Fc D122A groups, respectively. Interestingly, heart mass was not significantly changed by treatment. The average gastrocnemius myofiber cross-section area was higher in mice treated with AAV9-GDF11PRO-Fc (+ 23%; *p* = 0.0226) and AAV9-GDF11PRO-Fc D122A (+ 25%; *p* = 0.0170; Fig. 5c and d). In the MFD distribution analysis, MFD tended to peak around 20–30 μm across all the groups, which is likely due to the higher proportion of small regenerating myofibers in dystrophic muscle. However, treatment with AAV9-GDF11PRO-Fc and AAV9-GDF11PRO-Fc D122A did result in an increased proportion of myofibers with MFDs higher than 64 μm, suggesting that factor expression had induced myofiber hypertrophy (Fig. 5e) in skeletal muscle.

Western blot analysis confirmed protein expression of the transgenes in liver and serum. As expected, the band at 58 kDa corresponding to full-length GDF11PRO-Fc or GDF11PRO-Fc D122A was detectable in treated mice. Serum levels of the full-length propeptide were slightly higher in mice treated with AAV9-GDF11PRO-Fc D122A than in those treated with AAV9-GDF11PRO-Fc, suggesting that serum persistence of the active propeptide was increased by the D122A mutation (Fig. 5f).

Overall, these results indicate systemic expression of GDF11PRO-Fc and GDF11PRO-Fc D122A increases skeletal muscle mass in dystrophic mdx mice and that this skeletal muscle hypertrophy is associated with improvements in grip strength and motor coordination. However, treatment did not seem to affect muscle endurance based upon treadmill running performance. Additionally, the D122A mutant appeared to be slightly more effective at increasing muscle mass and function, presumably due to improved serum persistence.

### Systemic GDF11PRO-Fc gene delivery does not reduce the dystrophic pathology in mdx mice

A number of studies have reported that blockade of TGF-β-ActRII-SMAD2/3 signaling, whether by MSTN inhibition or blockade of ActRII, may have therapeutic benefit in animal models of DMD [36–45]. To determine the impact of AAV9-GDF11PRO-Fc treatment on the dystrophic pathology, we performed histological analysis of limb muscles and the diaphragm.

**Fig. 6:**
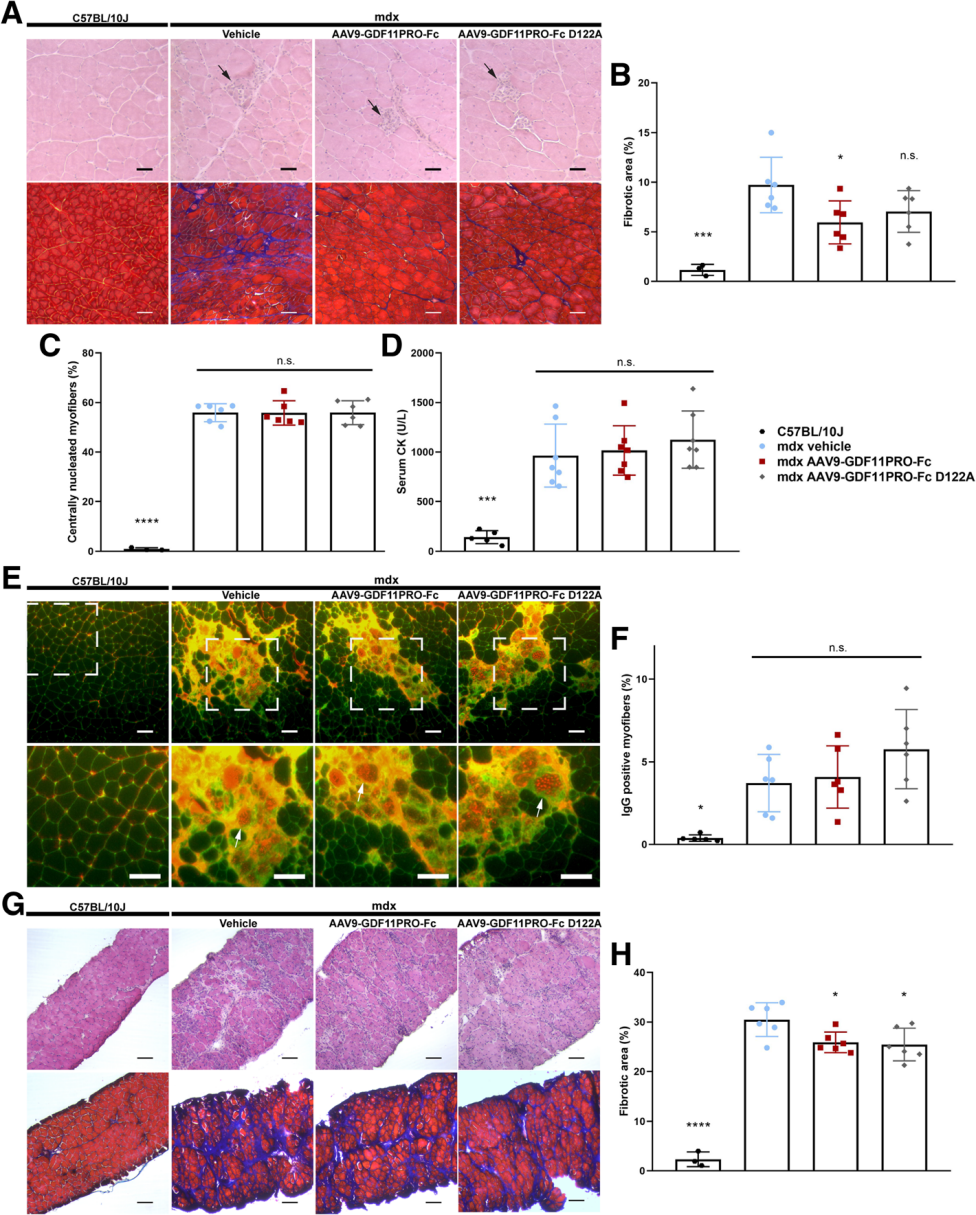
GDF11PRO-Fc does not mitigate the dystrophic pathology in mdx mice. 6-week-old mdx mice were treated with AAV9-GDF11PRO-Fc (*n* = 7), AAV9-GDF11PRO-Fc D122A (*n* = 7) or vehicle (*n* = 7) via tail vein injection. Age-matched C57BL/10J (*n* = 5) mice were also included as a wild type control. Mice were euthanized 12 weeks post-treatment. **a** Representative HE and MTC staining from gastrocnemius cross-sections of C57BL/10J and mdx mice. Central nucleation is evident in all the mdx groups. Localized areas of myofiber necrosis and regeneration are marked with a black arrow. Fibrotic area is represented by the blue region on MTC staining. Scale bars represent 50 μm in HE sections and 100 μm in MTC sections. **b** Fibrotic area in the gastrocnemius expressed as a percentage of the total muscle cross-section area. **c** Percentage of myofibers exhibiting central nucleation. **d** Measurement of circulating levels of serum CK, a marker of muscle damage. **e** Representative immunofluorescence images of mouse IgG staining (red) in gastrocnemius tissue cross-sections. Positive staining for IgG indicates myofiber permeability to serum proteins and loss of membrane integrity. Sections were co-stained with WGA to visualize individual myofibers. Zoomed-in area is marked with a white box. Characteristic IgG-positive myofibers are depicted with a white arrow. Scale bars represent 100 μm. **f** The area of IgG-positive myofibers expressed as a percentage of the total muscle cross-section area. **g** Representative HE and MTC staining from diaphragm cross-sections of C57BL/10J and mdx mice. Extensive pathology is apparent in mdx but not C57BL/10J mice. Scale bars represent 100 μm. **h** Fibrotic area in the diaphragm expressed as a percentage of the total cross-section area. *p < 0.05; ***p < 0.001; ****p < 0.0001; n.s. not significant; compared to vehicle-treated mdx controls

Characteristic signs of the dystrophic pathology, including muscle necrosis, central nucleation, intramuscular collagen deposition, and presence of inflammatory infiltrates were evident across all the mdx groups (Fig. 6a). In the gastrocnemius, the fibrotic area percentage was reduced by − 39% (*p* = 0.0273) in mice treated with AAV9-GDF11PRO-Fc. Fibrosis in the gastrocnemius was also slightly decreased in the AAV9-GDF11PRO-Fc D122A group; however, due to high intersubject variability in limb muscle intramuscular fibrosis, this difference did not reach statistical significance (− 28%; *p* = 0.1230; Fig. 6b). There was also no significant difference observed in the proportion of centrally nucleated myofibers, suggesting that myofiber regeneration was actively occurring across all the groups (Fig. 6c). Serum CK, a marker of muscle breakdown, was also not significantly changed by treatment (Fig. 6d). In addition, localized deterioration of myofiber membrane integrity, as measured by anti-mouse IgG immunofluorescence staining, was evident across all the mdx groups. Unexpectedly, mdx mice treated with AAV9-GDF11PRO-Fc D122A exhibited a slight increase in IgG-positive area on average, but this difference did not reach statistical significance (+ 55%; *p* = 0.1729; Fig. 6e and f; Additional file 1: Figure S5). Obvious signs of myofiber necrosis, regeneration, central nucleation, and IgG penetrance were not apparent in the wild type C57BL/10J gastrocnemius, indicating that these markers are specific to the dystrophic pathology (Fig. 6a–f; Additional file 1: Figure S5) [46].

In the diaphragm, H&E staining revealed extensive necrosis and intramuscular collagen deposition in both treated and untreated mdx groups (Fig. 6g). Similar to what was observed in the gastrocnemius muscle, treatment with AAV9-GDF11PRO-Fc or AAV9-GDF11PRO-Fc D122A produced a mild reduction in diaphragm intramuscular fibrosis overall. The average fibrotic area percentage in the diaphragm was reduced by 15% (*p* = 0.0328) and 17% (*p* = 0.019) with AAV9-GDF11PRO-Fc and AAV9-GDF11PRO-Fc D122A treatment, respectively (Fig. 6h). As expected, these pathological features were largely absent in the diaphragm of wild type C57BL/10J mice (Fig. 6g and h). From these data, we determine that GDF11PRO-Fc and GDF11PRO-Fc D122A are able to mildly inhibit intramuscular collagen deposition in the limb muscles and diaphragm over a 3-month period. However, treatment does not appear to halt the ongoing cycle of muscle degeneration and regeneration in the gastrocnemius or diaphragm of adult mdx mice.

## Discussion

It is well-established that ligands in the TGF-β superfamily have important roles in the regulation of many fundamental biological processes, including the maintenance of skeletal muscle homeostasis [47, 48]. The primary goal of the present study was to assess the effects of the GDF11 propeptide, which is an inhibitor of GDF11/MSTN, on skeletal muscle. To achieve this, we utilized AAV-mediated gene delivery to elicit long-term expression of a modified GDF11Pro-Fc construct in adult mice. Direct intramuscular administration of AAV into skeletal muscle may be used to achieve high levels of protein expression in the target tissue for local effect, although a considerable fraction of the vector dose may distribute into systemic circulation [49]. Intravenous AAV administration, on the other hand, can be used to achieve system-wide exposure primarily via AAV transduction of the liver and subsequent secretion of the desired protein product into circulation [35]. For in vivo experiments, we opted to use the AAV9 serotype, which has been shown to transduce the liver, heart, and skeletal muscle with high efficiency [34]. In addition, we selected the ubiquitous CAG promoter to elicit strong transgene expression from transduced cells. Through these measures, we are able to achieve high expression levels of the GDF11PRO-Fc protein with only a single vector administration.

Our findings from the pull-down assay show that GDF11PRO-Fc binds GDF11 and MSTN with high affinity. This result corroborates a previous study reporting that the activity of both GDF11 and MSTN are inhibited by pre-incubation with the GDF11 propeptide [21]. These results are not surprising given that the GDF11 and MSTN mature dimers are highly homologous in their protein sequences [1]. However, we find that GDF11PRO-Fc is not able to bind rActivin A, suggesting that the GDF11 propeptide has little to no activity against more distantly related TGF-β superfamily ligands. In C2C12 myotubes, AAV-mediated GDF11PRO-Fc expression reduces the magnitude of both rGDF11- and rMSTN-induced myotube atrophy, although GDF11PRO-Fc seems to have a stronger protective effect against rGDF11. Because the GDF11 propeptide is derived from the GDF11 precursor, it is possible that GDF11PRO-Fc has a higher affinity for GDF11 than MSTN. In adult mice, local expression of GDF11PRO-Fc induces muscle hypertrophy specifically at the site of injection, which suggests that GDF11PRO-Fc mediates its effects via blockade of GDF11/MSTN activity at ActRII on skeletal muscle. However, systemically-mediated mechanisms of muscle hypertrophy cannot be ruled out, as ActRII is expressed on many tissues throughout the body [50].

A number of studies have reported therapeutic benefit from inhibition of the TGF-β-ActRII-SMAD2/3 signaling axis in animal models of DMD [31, 36–41, 44, 45]. In mdx mice, the GDF11PRO-Fc D122A mutant appears to be slightly more effective at increasing skeletal muscle mass and improving skeletal muscle strength in comparison to the unmodified GDF11PRO-Fc, and this is likely due to increased serum persistence of the BMP1/TLD-like metalloproteinase-resistant D122A mutant. Indeed, a prior study reported that the half-life of a MPRO-Fc fusion protein administered by intraperitoneal injection could be extended from 2 h to 5–7 days with an analogous D76A mutation [51]. Because AAV-mediated gene delivery produces sustained transgene expression from non-dividing cells, it is likely that serum levels of the unmodified GDF11PRO-Fc still remain at effective concentrations due to ongoing secretion with the liver as a major site of protein production. The observation that peak grip strength improves with GDF11PRO-Fc treatment, but not running endurance, is supported by the findings that MSTN has a disproportionate effect on glycolytic type II fast-twitch muscle fibers [52, 53]. In addition, it has been shown that ActRII blockade in mdx mice induces a metabolic shift from β-oxidation to anaerobic glycolysis in skeletal muscle, leading to increased fatigability and exercise intolerance [54].

It has been reported that MSTN directly regulates muscle fibroblasts, and inhibition of MSTN inhibits fibrosis by inducing muscle fibroblast apoptosis [55, 56]. In mdx mice, we do observe a mild reduction in fibrosis following AAV9-GDF11PRO-Fc or AAV9-GDF11PRO-Fc D122A treatment. However, GDF11PRO-Fc treatment was not able to reduce other markers of the dystrophic pathology. We detect no significant difference in the proportion of centrally nucleated myofibers, serum CK or membrane permeability to IgG between the treatment groups, indicating that GDF11PRO-Fc or GDF11PRO-Fc D122A gene delivery is not able to halt the ongoing process of myofiber degeneration and regeneration. Actually, treatment with AAV9-GDF11PRO-Fc D122A appears to slightly increase the number of IgG-permeable myofibers in the gastrocnemius, suggesting a worsening of membrane integrity. A possible explanation for this unanticipated observation is that an increase in muscle strength induced by GDF11PRO-Fc exacerbates activity-induced muscle damage, as the absence of dystrophin renders myofibers highly susceptible to contraction-induced damage in dystrophic muscle [57, 58]. Indeed, an increased rate of contractures associated with muscular imbalances due to disproportionate muscle hypertrophy has been reported in a myostatin-deficient GRMD model [59]. Altogether, these results are not unforeseen, as GDF11PRO-Fc is not expected to correct the underlying cause of disease, which is the absence of functional dystrophin [60].

Although we show that GDF11PRO-Fc binds and blocks the activity of both GDF11 and MSTN, we predict that the in vivo effects observed in skeletal muscle are due primarily to blockade of circulating MSTN based on the finding that GDF11 levels are almost 500-fold lower than MSTN levels in mouse serum and therefore unlikely to outcompete MSTN for ActRII binding [59]. The anti-aging effects of GDF11 remain controversial [13]. Unfortunately, the effects of systemic GDF11 inhibition alone could not be delineated in this study because the evaluated factor, GDF11PRO-Fc, inhibits both GDF11 and MSTN. However, based upon our finding that GDF11 induces skeletal muscle atrophy in adult mice, it seems unlikely that GDF11 would be suitable as an effective anti-aging therapeutic for skeletal muscle dysfunction due to the substantial risk for muscle loss.

## Conclusions

We determine that GDF11PRO-Fc expression induces skeletal muscle hypertrophy and improvements in muscle strength in both normal and dystrophic mdx adult mice. In addition, GDF11PRO-Fc treatment mildly reduces intramuscular fibrosis in the skeletal muscle of mdx mice. However, GDF11PRO-Fc has no discernable effect on muscle necrosis, myofiber central nucleation, membrane integrity, or serum CK levels, suggesting that GDF11PRO-Fc treatment alone is not able to prevent progressive muscle deterioration in mdx mice.

## Additional file

Additional file 1: Supplementary manuscript figures (Figure S1, Figure S2, Figure S3, Figure S4, Figure S5). (PDF 2019 kb)

## Abbreviations

AAV: Adeno-associated virus
ActRII: Activin type II receptor
ALK: Type I activin receptor
BMP: Bone morphogenetic protein
CAG: Promoter consisting of cytomegalovirus enhancer, chicken β-actin promoter, and a rabbit β-globin intron
CsCl: Cesium chloride
D122A: Refers to aspartic acid to alanine mutation at amino acid 122
Fc: Fragment crystallizable
GDF11: Growth differentiation factor 11
GDF11PRO: GDF11 propeptide
GRMD: Golden retriever model of Duchenne muscular dystrophy
HE: Hematoxylin & eosin
ITR: Inverted terminal repeat
MFD: Minimum Feret diameter
MOI: Multiplicity of infection
MPRO: Myostatin propeptide
MSTN: Myostatin
MTC: Masson trichrome
PEG: Polyethylene glycol
qPCR: Quantitative polymerase chain reaction
TGF-β: Transforming growth factor β
TLD: Tolloid
