# Supplementary material for: A GDF11/myostatin inhibitor, GDF11 propeptide-Fc, increases skeletal muscle mass and improves muscle strength in dystrophic mdx mice
Source: Skelet Muscle. 2019 May 27;9:16. doi: 10.1186/s13395-019-0197-y (PMC6537384; doi:10.1186/s13395-019-0197-y)
Supplement: Supplementary file 1 — Supplementary manuscript figures (Figure S1, Figure S2, Figure S3, Figure S4, Figure S5). (PDF 2019 kb) [file 13395_2019_197_MOESM1_ESM.pdf]

A

GDF11PRO-Fc 5′-

ATGGTGCTGGCTGCTCCTCTGCTGCTGGGATTCCTGCTGCTGGCCCTGGAACTGAGGCCTAGAGGCGAAGCCGCTGAAGGACCTGCTGCCGCTGCCGCAGCCGCCGCTGCAG
CAGCAGCTGCAGGCGTTGGCGGAGAGAGATCTAGCAGACCTGCCCCTTCTGTGGCCCCTGAGCCTGATGGCTGTCCTGTGTGTGTGTGGCGGCAGCACAGCAGAGAGCTGCG
GCTGGAATCCATCAAGAGCCAGATCCTGAGCAAGCTGAGACTGAAAGAGGCCCCTAACATCAGCAGAGAGGTGGTCAAGCAGCTGCTGCCCAAGGCCCCTCCACTGCAGCAG
ATCCTGGACCTGCACGATTTCCAGGGCGACGCTCTGCAGCCCGAGGACTTCCTGGAAGAGGATGAGTACCACGCCACCACCGAGACAGTGATCAGCATGGCCCAAGAGACAG
ACCCCGCCGTGCAGACAGATGGCAGCCCTCTGTGCTGCCACTTCCACTTCAGCCCCAAAGTGATGTTCACCAAGGTGCTGAAGGCCCAGCTGTGGGTGTACCTGAGGCCTGT
GCCTAGACCCGCCACCGTGTATCTGCAGATCCTGCGGCTGAAGCCTCTGACCGGCGAGGGAACAGCTGGTGGCGGTGGCGGAGGACGGCGGCACATCAGAATCAGAAGCCTG
AAGATCGAGCTGCACTCCAGAAGCGGCCACTGGCAGAGCATCGACTTCAAACAGGTGCTGCACAGCTGGTTCCGGCAGCCCCAGAGCAACTGGGGCATCGAGATCAACGCCT
TCGACCCCAGCGGCACCGATCTGGCCGTTACATCTCTGGGACCTGGCGCCGAGGGCCTGCATCCCTTTATGGAACTGCGGGTGCTGGAAAACACCAAGGACAAGACCCACAC
CTGTCCTCCCTGCCCTGCCCCTGAACTGCTGGGCGGACCTAGCGTGTTCCTGTTCCCACCCAAGCCCAAGGACACCCTGATGATCAGCCGGACCCCTGAAGTGACCTGCGTG
GTGGTGGACGTGTCCCACGAGGATCCCGAAGTGAAGTTCAATTGGTACGTGGACGGCGTGGAAGTGCACAACGCCAAGACCAAGCCCAGAGAGGAACAGTACAACAGCACCT
ACCGGGTGGTGTCCGTGCTGACCGTGCTGCACCAGGACTGGCTGAACGGCAAAGAGTACAAGTGCAAGGTGTCCAACAAGGCCCTGCCTGCCCCAATCGAGAAAACCATCAG
CAAGGCCAAGGGCCAGCCCCGCGAGCCTCAGGTTTACACACTGCCTCCCAGCCGGGAAGAGATGACCAAGAACCAGGTGTCCCTGACATGCCTGGTCAAGGGCTTCTACCCC
AGCGATATCGCCGTGGAATGGGAGAGCAACGGCCAGCCTGAGAACAACTACAAGACCACCCCTCCCGTGCTGGACAGCGACGGCTCATTCTTCCTGTACAGCAAGCTGACCG
TGGACAAGAGCCGGTGGCAGCAGGGCAACGTGTTCAGCTGCTCTGTGATGCACGAGGCCCTGCACAACCACTACACCCAGAAGTCCCTGTCCCTGAGCCCCGGCAAGTGATG
A-3′

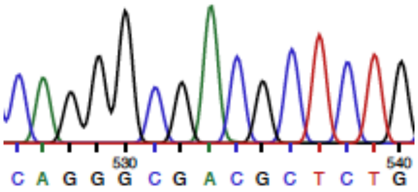

B

GDF11PRO-Fc D122A 5′-

ATGGTGCTGGCTGCTCCTCTGCTGCTGGGATTCCTGCTGCTGGCCCTGGAACTGAGGCCTAGAGGCGAAGCCGCTGAAGGACCTGCTGCCGCTGCCGCAGCCGCCGCTGCAG
CAGCAGCTGCAGGCGTTGGCGGAGAGAGATCTAGCAGACCTGCCCCTTCTGTGGCCCCTGAGCCTGATGGCTGTCCTGTGTGTGTGTGGCGGCAGCACAGCAGAGAGCTGCG
GCTGGAATCCATCAAGAGCCAGATCCTGAGCAAGCTGAGACTGAAAGAGGCCCCTAACATCAGCAGAGAGGTGGTCAAGCAGCTGCTGCCCAAGGCCCCTCCACTGCAGCAG
ATCCTGGACCTGCACGATTTCCAGGGCGCCGCTCTGCAGCCCGAGGACTTCCTGGAAGAGGATGAGTACCACGCCACCACCGAGACAGTGATCAGCATGGCCCAAGAGACAG
ACCCCGCCGTGCAGACAGATGGCAGCCCTCTGTGCTGCCACTTCCACTTCAGCCCCAAAGTGATGTTCACCAAGGTGCTGAAGGCCCAGCTGTGGGTGTACCTGAGGCCTGT
GCCTAGACCCGCCACCGTGTATCTGCAGATCCTGCGGCTGAAGCCTCTGACCGGCGAGGGAACAGCTGGTGGCGGTGGCGGAGGACGGCGGCACATCAGAATCAGAAGCCTG
AAGATCGAGCTGCACTCCAGAAGCGGCCACTGGCAGAGCATCGACTTCAAACAGGTGCTGCACAGCTGGTTCCGGCAGCCCCAGAGCAACTGGGGCATCGAGATCAACGCCT
TCGACCCCAGCGGCACCGATCTGGCCGTTACATCTCTGGGACCTGGCGCCGAGGGCCTGCATCCCTTTATGGAACTGCGGGTGCTGGAAAACACCAAGGACAAGACCCACAC
CTGTCCTCCCTGCCCTGCCCCTGAACTGCTGGGCGGACCTAGCGTGTTCCTGTTCCCACCCAAGCCCAAGGACACCCTGATGATCAGCCGGACCCCTGAAGTGACCTGCGTG
GTGGTGGACGTGTCCCACGAGGATCCCGAAGTGAAGTTCAATTGGTACGTGGACGGCGTGGAAGTGCACAACGCCAAGACCAAGCCCAGAGAGGAACAGTACAACAGCACCT
ACCGGGTGGTGTCCGTGCTGACCGTGCTGCACCAGGACTGGCTGAACGGCAAAGAGTACAAGTGCAAGGTGTCCAACAAGGCCCTGCCTGCCCCAATCGAGAAAACCATCAG
CAAGGCCAAGGGCCAGCCCCGCGAGCCTCAGGTTTACACACTGCCTCCCAGCCGGGAAGAGATGACCAAGAACCAGGTGTCCCTGACATGCCTGGTCAAGGGCTTCTACCCC
AGCGATATCGCCGTGGAATGGGAGAGCAACGGCCAGCCTGAGAACAACTACAAGACCACCCCTCCCGTGCTGGACAGCGACGGCTCATTCTTCCTGTACAGCAAGCTGACCG
TGGACAAGAGCCGGTGGCAGCAGGGCAACGTGTTCAGCTGCTCTGTGATGCACGAGGCCCTGCACAACCACTACACCCAGAAGTCCCTGTCCCTGAGCCCCGGCAAGTGATG
A-3′

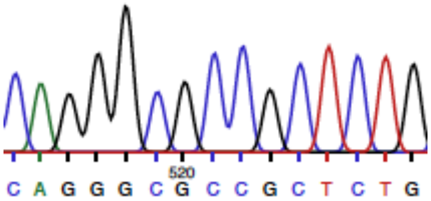

C

GDF11 5′-

ATGGTGCTGGCTGCCCCCCTGCTGCTGGGATTTCTGCTGCTGGCTCTGGAACTGCGGCCTAGAGGCGAAGCCGCTGAAGGACCAGCTGCCGCTGCTGCTGCCGCAGCCGCCG
CAGCTGCAGCAGGCGTGGGCGGAGAAAGAAGCTCTAGACCTGCCCCTAGCGTGGCCCCTGAGCCTGATGGATGTCCTGTGTGCGTGTGGCGGCAGCACTCCAGAGAGCTGCG
GCTGGAATCCATCAAGAGCCAGATCCTGAGCAAGCTGAGACTGAAAGAGGCCCCCAACATCAGCCGCGAGGTCGTGAAACAGCTGCTGCCCAAAGCCCCCCCACTGCAGCAG
ATTCTGGACCTGCACGACTTCCAGGGGGACGCCCTGCAGCCCGAGGATTTCCTGGAAGAGGACGAGTACCACGCCACCACCGAGACAGTGATCAGCATGGCCCAGGAAACCG
ACCCCGCCGTGCAGACAGATGGCAGCCCTCTGTGCTGCCACTTCCACTTCAGCCCCAAAGTGATGTTCACCAAGGTGCTGAAAGCCCAGCTGTGGGTGTACCTGAGGCCCGT
GCCTAGACCAGCCACCGTGTACCTGCAGATCCTGCGGCTGAAGCCTCTGACCGGCGAGGGAACAGCTGGCGGCGGAGGGGGAGGAAGAAGGCACATCAGAATCAGAAGCCTG
AAGATCGAGCTGCACAGCAGAAGCGGCCACTGGCAGAGCATCGACTTCAAACAGGTGCTGCACTCCTGGTTCCGGCAGCCCCAGAGCAATTGGGGCATCGAGATCAACGCCT
TCGACCCCAGCGGCACCGATCTGGCCGTGACATCTCTGGGACCTGGCGCCGAAGGCCTGCACCCCTTCATGGAACTGAGAGTGCTGGAAAACACCAAGCGGAGCCGGCGGAA
CCTGGGCCTGGATTGTGATGAGCACAGCAGCGAGAGCCGGTGCTGCAGATACCCCCTGACCGTGGACTTCGAGGCCTTTGGCTGGGACTGGATCATTGCCCCCAAGCGGTAC
AAGGCCAACTACTGCAGCGGCCAGTGCGAGTACATGTTCATGCAGAAGTACCCCCACACCCACCTGGTGCAGCAGGCCAATCCTAGAGGCTCTGCCGGCCCTTGCTGCACCC
CCACAAAGATGAGCCCCATCAACATGCTGTACTTCAACGACAAGCAGCAGATCATCTACGGCAAGATCCCCGGCATGGTGGTGGACAGATGCGGCTGCTCCTGATGA-3′

Figure S1. Entire DNA sequences of transgenes. (A) Sequence of GDF11PRO-Fc with D122 position in blue with the Sanger sequencing output of the highlighted region. **(B)** Sequence of GDF11PRO-Fc D122A with mutated site in red with the Sanger sequencing output of the highlighted region. **(C)** Sequence of GDF11.

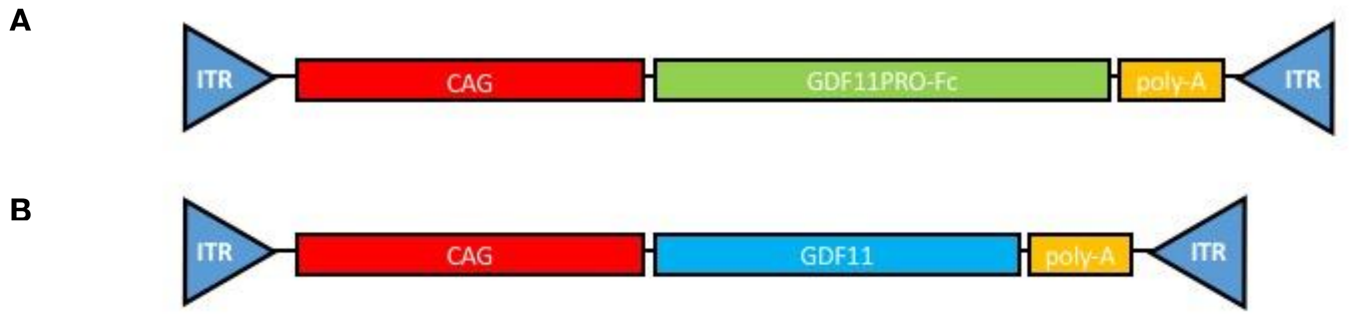

Figure S2. Vector construction. (A) GDF11PRO-Fc transgene cassette. **(B)** GDF11 transgene cassette. ITR: AAV2 inverted terminal repeat; CAG: promoter, containing the cytomegalovirus (CMV) early enhancer, first exon and first intron of chicken β-actin gene and splice acceptor of rabbit β-globin; poly-A: bGH (bovine growth hormone) polyadenylation signal

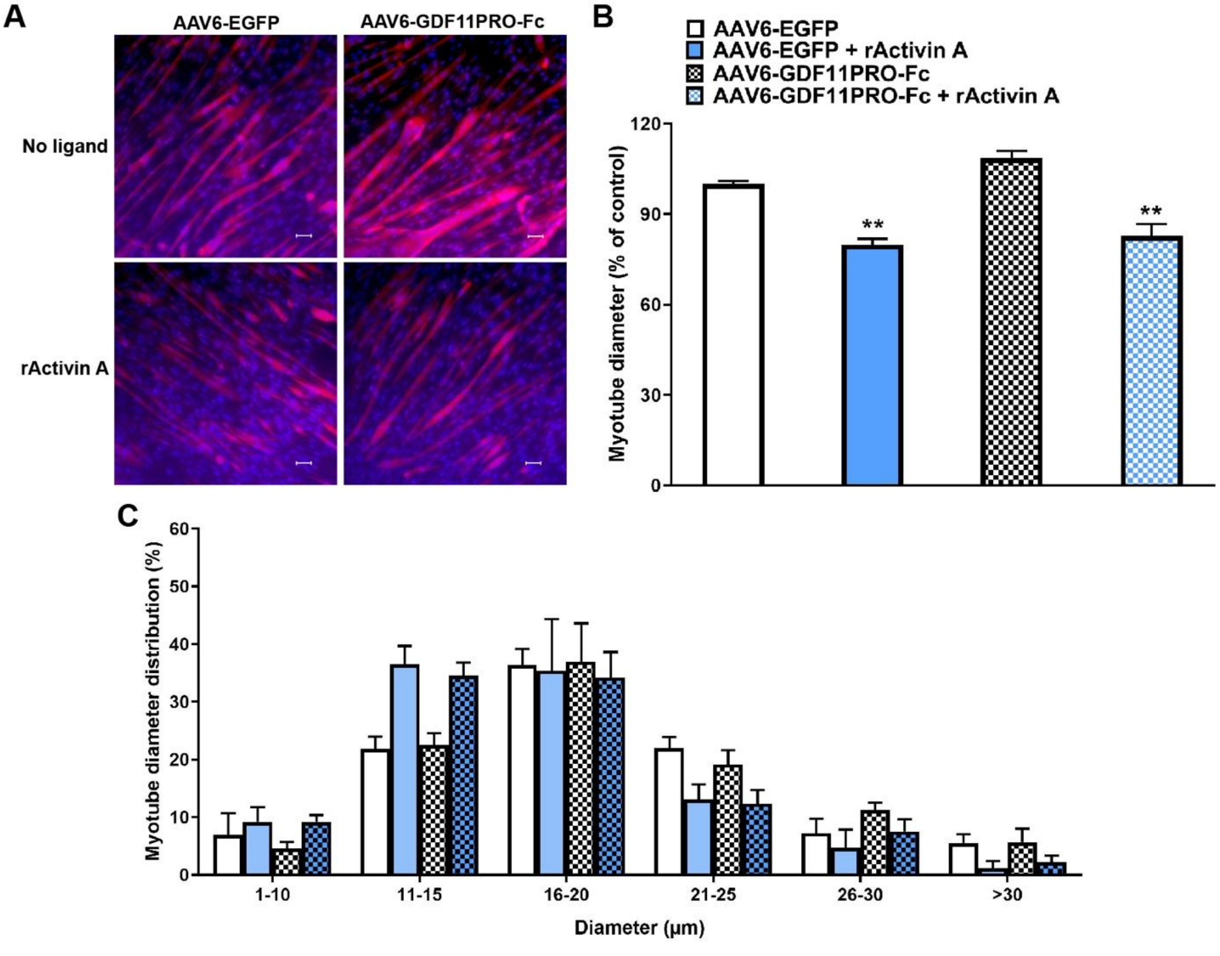

Figure S3. GDF11PRO-Fc does not prevent rActivin A-induced myotube atrophy in C2C12 cells. Differentiated C2C12 myotubes were infected with AAV6-EGFP (control) or AAV6-GDF11PRO-Fc. **(A)** Representative immunofluorescence images of dystrophin-stained C2C12 myotubes 72 hours after treatment with rActivin A or nothing. Scale bar represents 50 µm. **(B)** Myotube diameter relative to control. **(C)** Distribution of myotube diameter. **$p < 0.01$

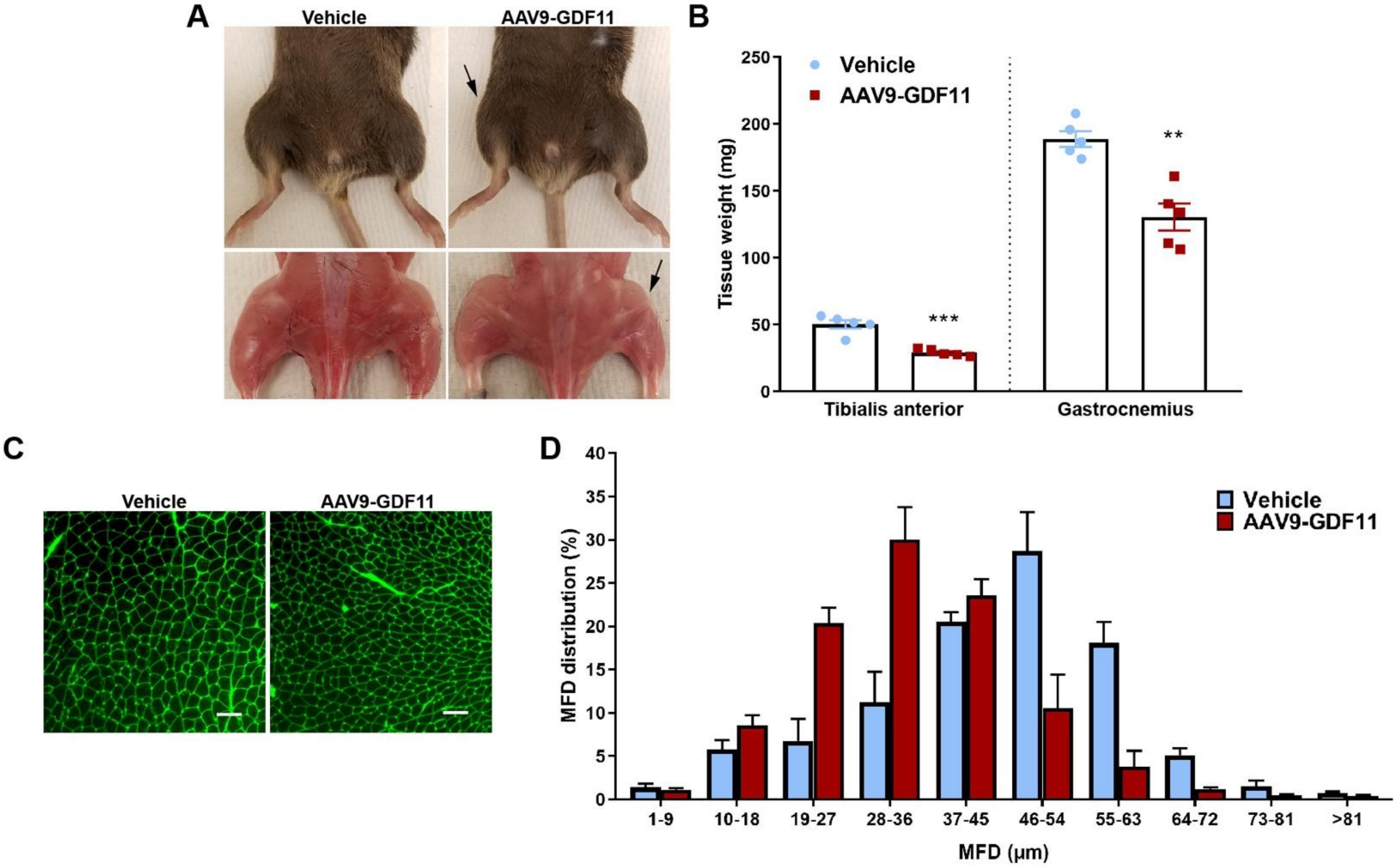

Figure S4. GDF11 overexpression induces muscle atrophy after localized intramuscular injection. 8-week-old male C57BL/6J mice were administered a unilateral intramuscular injection of AAV9-GDF11 or vehicle at a dose of $1x10^{12}$ vg/kg ($2.5x10^{10}$ vg/mouse) into the right-side hindlimb. Mice were sacrificed 10 weeks post-injection. **(A)** Gross musculature of mice hindlimbs. Muscle atrophy in the AAV9-GDF11-injected limb was evident (indicated by black arrow). **(B)** Wet tissue mass of injected right-side tibialis anterior and gastrocnemius. **(C)** Representative immunofluorescence images of AlexaFluor-488-conjugated wheat germ agglutinin-stained sections of the injected right-side gastrocnemius. Scale bar represents 100 µm. **(D)** Minimum Feret diameter (MFD) distribution of muscle fibers in the injected-right side gastrocnemius. ***$p < 0.001$; **$p < 0.01$

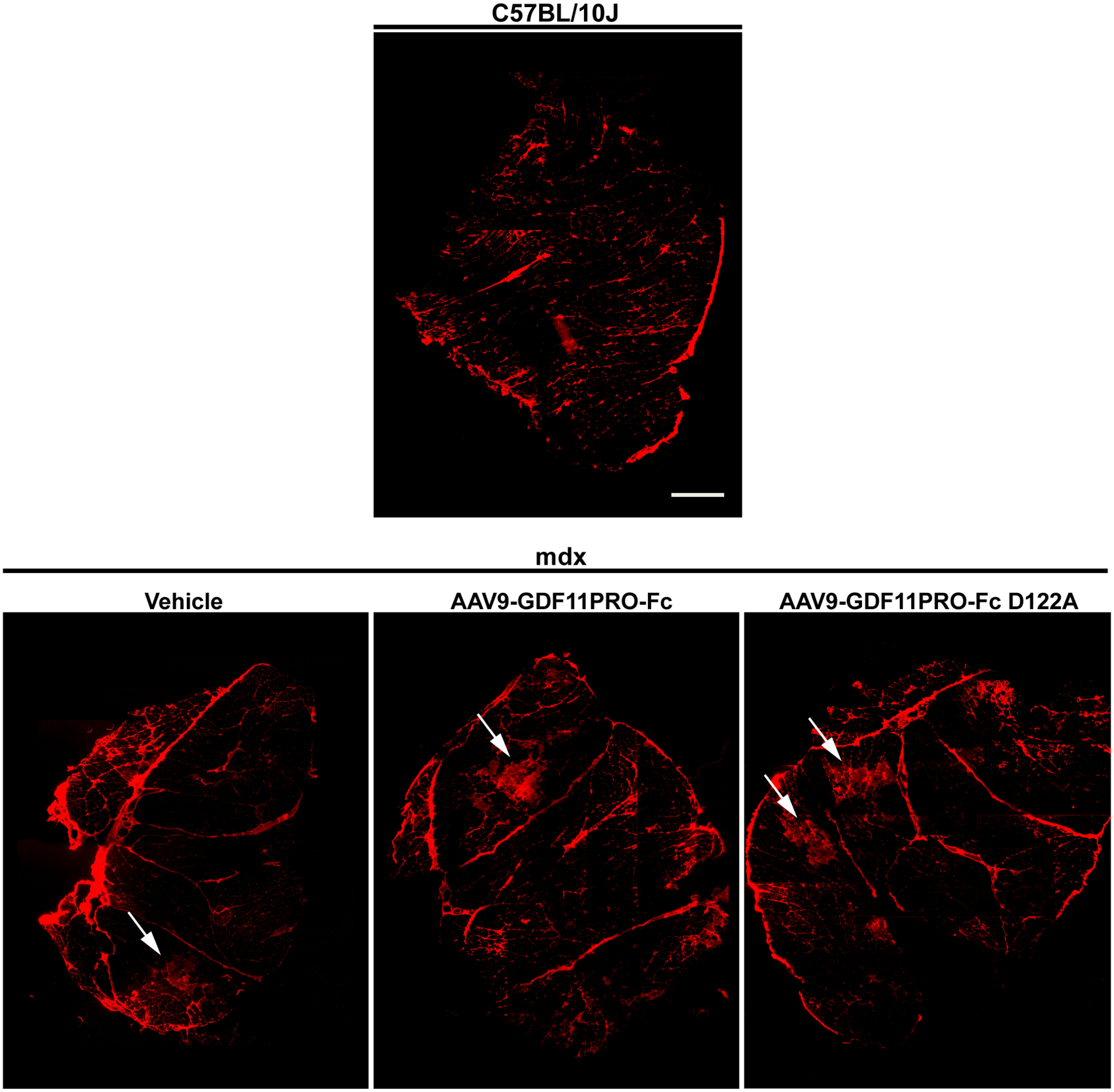

Figure S5. Serum IgG permeability in mdx gastrocnemius muscle. 6-week old male mdx mice were treated with a single intravenous injection of AAV9-GDF11PRO-Fc, AAV9-GDF11PRO-Fc D122A or vehicle at a dose of $3x10^{13}$ vg/kg ($1x10^{12}$ vg/mouse). Age-matched male C57BL/10J mice were used as a wild type control. Mice were sacrificed 12 weeks post-injection. Transverse gastrocnemius muscle sections were collected and stained with a Cy3-conjugated anti-mouse IgG antibody for immunofluorescence analysis. Up to 30 separate images per sample were stitched together using a semi-automated approach to generate the final composite. Regions of positive myofiber IgG staining are marked with white arrows. Scale bar represents 300 µm.
